# Assessment of Antioxidant Capacity, Phytochemical Phenolic Composition, Cholinesterase and α-Glucosidase Inhibitory Activities, and Antimicrobial Potential of *Tribulus terrestris*

**DOI:** 10.3390/molecules31193370

**Published:** 2026-09-22

**Authors:** Ashwell R. Ndhlala, Emrah Dikici, Sevgi Altın, Arzu Kavaz Yüksel, Mesut Işık, Ekrem Köksal

**Affiliations:** 1 Green Biotechnologies Research Centre, School of Agricultural and Environmental Sciences, University of Limpopo, Private Bag X1106, Polokwane 0727, South Africa; 2 Dogubayazit Ahmed-i Hani Vocational School, Agri Ibrahim Cecen University, Agri 04100, Türkiye; 3 Central Researching Laboratory, Agri Ibrahim Cecen University, Agri 04100, Türkiye; 4 Department of Chemistry, Faculty of Science and Art, Erzincan Binali Yıldırım University, Erzincan 24100, Türkiye; 5 Department of Food Technology, Technical Sciences Vocational School, Atatürk University, Erzurum 25030, Türkiye; 6 Department of Bioengineering, Faculty of Engineering, Bilecik Şeyh Edebali University, Bilecik 11230, Türkiye

**Keywords:** *Tribulus terrestris*, phytochemical analysis, antioxidant, acetylcholinesterase, lipid peroxidation, antimicrobial, LC-MS/MS, antimicrobial activity, antioxidant activity

## Abstract

*Tribulus terrestris* (TT) is a medicinal plant traditionally utilized in the management of various health conditions, including inflammation, edema, and hypertension. The present study aimed to investigate the phenolic profile of the ethanolic extract of TT and to evaluate its antioxidant, antimicrobial, anticholinesterase, and α-glucosidase inhibitory properties. Phenolic analysis revealed several quantified compounds, including vanillic acid, resveratrol, fumaric acid, acetohydroxamic acid, quercetin, hydroxybenzoic acid, salicylic acid, butein, caffeic acid, and catechin hydrate, while phloridzin dihydrate was detected below the quantification limit. Among the detected compounds, vanillic acid was found to be the predominant phenolic component. Biological activity assays demonstrated that the extract possessed remarkable cholinesterase inhibitory potential, exhibiting IC_50_ values of 9.11 ± 2.12 μg mL^−1^ and 8.77 ± 0.62 μg mL^−1^ against acetylcholinesterase (AChE) and butyrylcholinesterase (BChE), respectively. Furthermore, antioxidant capacity was confirmed through FRAP and CUPRAC assays, with corresponding values of 0.136 and 0.296. The antimicrobial efficacy of the extract was evaluated against *Staphylococcus aureus*, *Escherichia coli*, and *Salmonella Typhimurium*, revealing inhibitory effects against all tested bacterial strains. Overall, the findings indicate that the TT represents a promising natural source of phenolic compounds with significant antioxidant, antibacterial, and enzyme inhibitory activities, supporting its potential application in the development of pharmaceutical and therapeutic products.

## 1. Introduction

*Tribulus terrestris* (TT), the sole representative of its genus in Anatolia, belongs to the *Zygophyllaceae* family and is characterized as a small, prostrate annual herb. The species is widely distributed across tropical, subtropical, Mediterranean, and arid regions, including Europe, Asia, Africa, and North America, with documented occurrences in countries such as China, India, Spain, Bulgaria, and Türkiye [1,2,3]. Owing to its remarkable ecological adaptability, TT commonly colonizes arid and semi-arid habitats, particularly sandy soils, where plant diversity is relatively limited. Under shaded environmental conditions, the plant exhibits increased vertical growth, making it more conspicuous than surrounding vegetation [1]. TT is one of the most widely distributed and economically important species within the genus *Tribulus*. It is known by various vernacular names, including puncture vine, puncture clover, caltrop, goat’s head, bull’s head, ground burr nut, devil’s thorn, Bhakra, and Gokhru [4,5]. In Arabic-speaking countries, it is also referred to as Al-Gutub, Qutiba, Hasak, and Ders El-Agouz [6].

The TT has long been utilized in traditional medical systems, particularly in Traditional Chinese Medicine, for the treatment of various conditions. Traditionally, it has been employed as a tonic, aphrodisiac, diuretic, antihypertensive, analgesic, and urinary antiseptic agent [7,8]. Its therapeutic value has been linked to a rich phytochemical profile containing steroidal saponins, flavonoids, alkaloids, phenolics, phytosterols, and other secondary metabolites [5,8].

Experimental and clinical studies have demonstrated the pharmacological potential of TT in multiple physiological systems, including the reproductive, cardiovascular, nervous, gastrointestinal, and urinary systems [9,10,11,12]. Particular attention has focused on its beneficial effects on male reproductive function, such as improvements in testosterone levels, sperm quality, and fertility parameters [13,14]. In addition, TT has been investigated for its potential role in the management of diabetes, hypertension, hypercholesterolemia, cardiovascular disorders, and hepatic and renal diseases [15,16,17,18,19]. The plant also exhibits diverse biological activities, including antioxidant, anti-inflammatory, antimicrobial, antitumor, and antiproliferative effects [9,10,11,12,20,21,22,23].

The medicinal importance of TT is further supported by its strong antioxidant potential. Oxidative stress, resulting from an imbalance between reactive oxygen and nitrogen species and endogenous antioxidant defenses, contributes to cellular damage through lipid peroxidation, protein oxidation, and DNA impairment [5,24,25,26]. This process plays a significant role in the development of chronic diseases such as diabetes, cardiovascular disorders, cancer, neurodegenerative diseases, and obesity-related metabolic complications [26,27,28]. Consequently, natural antioxidant sources have attracted increasing scientific interest [29].

Steroidal saponins are considered the major bioactive constituents of TT and are largely responsible for its pharmacological activities [5,30]. Nevertheless, the concentration and composition of these compounds may vary according to geographical origin, environmental conditions, and cultivation factors, influencing the biological properties of the plant.

The rich phytochemical composition of TT has been associated with numerous biological activities, including antioxidant, antimicrobial, antiurolithiatic, hepatoprotective, anticancer, and endocrine-modulating effects [31,32,33,34,35,36]. In particular, oxidative stress has been closely linked to the pathogenesis of neurodegenerative disorders and diabetes mellitus. Consequently, inhibition of acetylcholinesterase (AChE) and α-glucosidase has emerged as an important therapeutic strategy for the management of Alzheimer’s disease and postprandial hyperglycemia, respectively. Plant-derived phenolic compounds and other secondary metabolites have attracted considerable interest as potential sources of multifunctional agents exhibiting both antioxidant and enzyme-inhibitory activities.

Despite the extensive pharmacological investigations conducted on TT, comprehensive studies integrating phytochemical characterization with antioxidant, enzyme-inhibitory, and antimicrobial evaluations remain limited. Furthermore, information regarding the relationship between phenolic composition and the biological activities of ethanolic extracts is still insufficient.

Therefore, the present study aimed to characterize the phenolic profile of TT ethanolic extract using LC–MS/MS analysis and to evaluate its antioxidant potential through radical scavenging and metal-reducing assays. Additionally, acetylcholinesterase and α-glucosidase inhibitory activities were investigated to assess potential neuroprotective and antidiabetic properties, while antimicrobial activity was evaluated through the determination of minimum inhibitory concentration (MIC) values against selected microbial strains.

## 2. Results and Discussion

### 2.1. Quantitative Assessment of Phytochemical Phenolic Constituents Using the LC–MS/MS Analytical Technique

Reactive oxygen species (ROS) and free radicals are continuously generated in living cells as byproducts of normal biochemical and physiological processes. However, when produced in excess, these reactive molecules play a major role in the onset and progression of a wide range of chronic diseases, including cancer, diabetes, age-associated disorders, and other degenerative conditions, mainly through oxidative damage to cellular macromolecules such as proteins, lipids, and DNA [37,38].

In the proposed LC–MS/MS method, the linearity of each analyte was evaluated by analyzing standard calibration solutions prepared over the concentration range of 1–2000 µg L^−1^. All analytes showed acceptable linearity within this working range. The corresponding regression equations and correlation coefficients are presented in Table 1. The R^2^ values of the calibration curves were higher than 0.99 for all analytes, indicating that the method exhibited suitable linearity for the quantitative determination of the investigated phenolic compounds. The LOD and LOQ values obtained using the analytical method ranged from 0.1 to 142.3 μg L^−1^ and from 0.3 to 274.6 μg L^−1^, respectively. In general, the recovery values of the phenolic compounds ranged from 96.6% to 101.1%. Recovery values were calculated as the ratio of the measured concentration to the spiked theoretical concentration multiplied by 100 and are presented as percentages in Table 1. These results indicate that the analytical method provided acceptable accuracy and reliability for the quantitative determination of the investigated phenolic compounds. Comparison with previously published method validation studies for the determination of phenolic compounds demonstrated that the results obtained in the present study were consistent with those reported in the literature [39]. Furthermore, the calculated R^2^ values were found to fall within the ranges previously reported in related studies.

Furthermore, the obtained R^2^ values were determined to be within the ranges reported in the existing literature. These findings are in agreement with the results obtained in the present study. The phenolic composition of TT, including flavonoids and phenolic acids, was successfully characterized in accordance with established analytical standards. According to Table 1, the predominant compound identified in TT was vanillic acid (1080.74 μg L^−1^). In a previous LC–MS/MS-based study, Sofi et al. [40] reported vanillin as one of the major phenolic constituents in the TTextract. In the present study, vanillic acid was the predominant compound reliably quantified in the TT ethanolic extract. Vanillic acid and vanillin are chemically distinct compounds and should not be directly compared as equivalent analytes. Nevertheless, these compounds are structurally related phenolic derivatives, and differences in the phenolic profile may be associated with geographical and ecological conditions, possible chemotypic variation, collection period, post-harvest processes, extraction conditions, analytical methodology, or the possible oxidative conversion of vanillin to vanillic acid [41,42]. As shown in Table 1, vanillic acid was the predominant compound reliably quantified in the TT extract, with a concentration of 1080.74 µg L^−1^. It was followed, in descending order, by resveratrol, fumaric acid, acetohydroxamic acid, quercetin, 4-hydroxybenzoic acid, salicylic acid, butein, caffeic acid, and catechin hydrate. Although chromatographic signals corresponding to phloridzin dihydrate and kaempferol were detected, their responses were below the corresponding LOQ values; therefore, both compounds were reported as DNQ and were not included in the quantitative ranking or comparative interpretation of the identified compounds. The obtained results demonstrated that the phenolic profile of the TT ethanolic extract was characterized by a diverse composition of flavonoids and phenolic acids. In contrast, eight different compounds presented in the table were not detected in the composition of TT. The concentrations obtained in the present study showed a decreasing trend from 1080.74 μg L^−1^ to 25.76 μg L^−1^. The identified compounds were primarily composed of phenolics, flavonoids, glycosides, phenolic acids, and their derivatives. The phenolic composition and bioactive characteristics of TT are affected by various factors, including cultivar differences, cultivation conditions, fruit size, floral characteristics, and harvesting time.

The outcomes of the present investigation are in agreement with the findings of Sofi et al. [40], who reported comparable effects following the application of *Tribulus terrestris* extract. Also, phytochemical investigations have identified flavonoids, alkaloids, α-amyrin, and saponins as the major bioactive compounds present in the TT [43,44]. The composition and relative abundance of these constituents may vary considerably depending on factors such as the geographical source of the plant material, the extraction procedures employed, and the solvent systems utilized during the extraction process.

Representative LC–MS/MS chromatograms of the identified compounds, with the corresponding peaks clearly labeled, are provided in the Supplementary Materials (Figure S1) to support compound assignment and chromatographic interpretation.

### 2.2. Antioxidant Activities of Tribulus terrestris

Variations in the phenolic composition of plants have been attributed to differences in their structural characteristics. Previous studies have indicated that the strong relationship observed between phenolic constituents and antioxidant activity supports the view that phenolic compounds present in plant extracts play a substantial role in overall antioxidant capacity [45]. Furthermore, the health-promoting effects associated with vegetables, fruits, and medicinal plants are largely linked to their elevated antioxidant potential [46]. DPPH assay is widely employed to evaluate the free radical scavenging capacity of antioxidant compounds. This method is based on the decrease in absorbance of an alcoholic DPPH solution following interaction with hydrogen-donating antioxidants [47]. In contrast, the ABTS assay involves the generation of the ABTS radical cation through the oxidation of ABTS^2−^ by potassium persulfate (K_2_S_2_O_8_), and the resulting radical activity is quantified using spectrophotometric techniques [48].

The free radical scavenging potential of TT ethanol extracts was evaluated using DPPH and ABTS methods. As presented in Table 2, the ethanol extract at a concentration of 0.2 μg mL^−1^ showed approximately 6.2% DPPH and 10.4% ABTS radical scavenging activity. In the CUPRAC and Fe^3+^–Fe^2+^ reduction tests regarding metal reduction capacity, the highest activity was determined in Trolox (0.516 at 0.2 μg mL^−1^), and it was followed by TT (0.296 at 0.2 μg mL^−1^) (Table 2). Although the metal reduction capacity of the plant extract was lower compared to Trolox, it appears that the phenolic compounds it contains provide moderate metal reduction and radical scavenging activity. Although the antioxidant activity of the plant extract was found to be lower than that of standard antioxidants, it is noteworthy due to its natural origin and potential economic value. These results may be due to the low amount of antioxidant compounds in the extract. DPPH scavenging data indicate that the extract has the ability to scavenge free radicals; furthermore, it is thought that this herbal product, as a natural antioxidant source, may limit the initiation and propagation of free radical-mediated chain reactions.

### 2.3. Anticholinergic and Antidiabetic Potential

#### Enzyme Activity

The inhibitory effects of TT ethanol extract on AChE, BChE, and α-glucosidase enzymes were investigated. Graphs of the results are presented in Figure 1, and IC_50_ values are presented in Table 3.

The findings in Table 3 clearly demonstrate that the standard inhibitor, tacrine, exhibited the strongest inhibitory activity against AChE and BChE among the tested samples. Tacrine showed the lowest IC_50_ values for both enzymes (AChE: 3.89 ± 0.28 μg mL^−1^; R^2^ = 0.998; BChE: 3.36 ± 0.32 μg mL^−1^; R^2^ = 0.959), confirming its high inhibitory capacity. In turn, TT ethanol extract also exhibited inhibitory activity against both cholinesterase enzymes, with IC_50_ values of 9.11 ± 2.12 μg mL^−1^ for AChE (R^2^ = 0.992) and 8.77 ± 0.62 μg mL^−1^ for BChE (R^2^ = 0.972). Although the inhibitory activity of TT extract was lower than that of tacrine, the extract demonstrated measurable cholinesterase inhibition under the tested experimental conditions.

Regarding α-glucosidase inhibition, acarbose showed the highest inhibitory activity with an IC_50_ value of 4.00 ± 0.35 μg mL^−1^ (R^2^ = 0.998). The TT ethanol extract also exhibited considerable α-glucosidase inhibition, with an IC_50_ value of 8.45 ± 0.45 μg mL^−1^ (R^2^ = 0.989). These findings indicate that TT ethanol extract possesses inhibitory effects against both cholinergic and carbohydrate-metabolizing enzymes, supporting further investigation of its phytochemical composition and biological properties.

Alzheimer’s disease (AD) is the most common cause of dementia and is characterized by progressive neurodegeneration and cognitive decline. Since cholinergic dysfunction is closely associated with memory impairment, inhibition of AChE and BChE remains one of the major approaches investigated in AD-related enzyme studies [49,50]. Phenolic acids and flavonoids found in medicinal and aromatic plants have been widely studied due to their diverse biological activities, including enzyme inhibitory properties [51,52]. In the present study, LC-MS/MS analysis revealed that TT ethanol extract contains several phenolic and related compounds, including vanillic acid, resveratrol, fumaric acid, acetohydroxamic acid, quercetin, hydroxybenzoic acid, and salicylic acid.

Several compounds identified in TT extracts have previously been investigated for their enzyme-inhibitory properties. Quercetin and resveratrol have been reported to exhibit cholinesterase and carbohydrate-metabolizing enzyme inhibitory activities in isolated compound studies, while vanillic acid and hydroxybenzoic acid derivatives have also been associated with enzyme inhibition [53,54,55,56,57,58,59,60,61]. These findings suggest that the phytochemical composition of TT extract may contribute to the observed enzyme inhibition.

The cholinesterase inhibitory activity observed in the present study is consistent with previous findings reported for TT extracts. Orhan et al. [62], Ghareeb et al. [63], and Uysal et al. [64] demonstrated AChE and BChE inhibitory effects in different TT preparations. Similarly, Kaya et al. [51] compared TT extracts obtained using different solvents and reported that the ethanol extract exhibited the strongest inhibitory activity, with IC_50_ values of 12.13 μg mL^−1^ for AChE and 22.33 μg mL^−1^ for BChE. In comparison, the IC_50_ values obtained in the present study (9.11 ± 2.12 μg mL^−1^ for AChE and 8.77 ± 0.62 μg mL^−1^ for BChE) indicate a comparable or higher inhibitory capacity under the tested experimental conditions. Differences among studies may be related to extraction methods, solvent properties, phytochemical variation, and assay conditions.

A similar trend was observed for α-glucosidase inhibition. The TT ethanol extract exhibited a dose-dependent inhibitory effect, with an IC_50_ value of 8.45 ± 0.45 μg mL^−1^. Previous studies have also reported α-glucosidase inhibitory activity for TT extracts. Lamba et al. [65] demonstrated α-glucosidase inhibition by TT ethanol extract, whereas Ercan and El [66] reported a considerably higher IC_50_ value of 6967 ± 343 μg mL^−1^. The lower IC_50_ value obtained in the present study suggests a strong inhibitory effect of the tested extract compared with some previously reported TT preparations.

The presence of phenolic constituents, including quercetin and hydroxybenzoic acid derivatives identified in TT extracts, may partly contribute to the observed α-glucosidase inhibitory activity, as several phenolic compounds and plant-derived polyphenols have been reported to interfere with carbohydrate-metabolizing enzymes [57,58,59,61,67,68,69,70,71]. However, the inhibitory activity of a crude extract represents the combined contribution of multiple bioactive constituents, and the specific contribution of individual compounds cannot be established without further isolation and mechanistic studies. In agreement with this concept, Song et al. [72] reported potent α-glucosidase inhibitory activity of cinnamic acid amide derivatives isolated from TT, with N-trans-coumaryltyramine exhibiting an IC_50_ value of 0.42 µM. Furthermore, AlKhaldi et al. [73] demonstrated that the extraction solvent significantly affects the α-glucosidase inhibitory potential of TT extracts, reporting an IC_50_ value of 27.28 µg/mL for the hexane extract. Collectively, these findings suggest that the α-glucosidase inhibitory activity of TT may arise from synergistic interactions among diverse phytochemical constituents rather than a single compound.

Overall, the present study demonstrates that TT ethanol extract exhibits notable in vitro inhibitory effects against AChE, BChE, and α-glucosidase enzymes. The observed activities may be associated with the complex phytochemical profile of the extract, including phenolic compounds identified by LC-MS/MS analysis. However, the present findings do not allow direct attribution of these effects to specific compounds. Future studies involving fractionation, isolation of active constituents, and mechanistic approaches may provide further insight into the compounds and interactions contributing to the observed enzyme inhibitory properties.

### 2.4. Antimicrobial Potential of Tribulus terrestris

Plants with medicinal properties have been collected and used by humans for centuries to treat various diseases, both worldwide and across different regions of our country. In recent years, despite significant advances in technology and modern medicine, there has been a continued preference for natural products, largely due to their perceived safety and health benefits. In this study, the antimicrobial activity of the extract derived from TT was evaluated against three microbial strains, namely *Staphylococcus aureus*, *Escherichia coli*, and *Salmonella typhimurium*, using both the disk diffusion assay and the minimum inhibitory concentration (MIC) method. Ciprofloxacin served as the reference standard for assessing antimicrobial efficacy (Table 4 and Table 5). An examination of Table 4 revealed that the plant extract at a concentration of 312 μg/mL produced inhibition zones of 8 mm against *S. typhimurium*, 5 mm against *E. coli*, and 4 mm against *S. aureus*. Nevertheless, the extract’s antimicrobial activity was considerably lower than that of Ciprofloxacin. Evaluation of the MIC results demonstrated that the minimum inhibitory concentration, defined as the lowest concentration of the extract that completely prevents visible microbial growth [74,75], was determined to be 312 μg/mL for all tested strains (Table 5).

The results indicated that the investigated plant extract exhibited relatively weak antimicrobial activity against the tested microorganisms, with inhibition zones ranging only from 4 to 8 mm. This activity is considerably lower than that of the reference antibiotic, Ciprofloxacin (18–21 mm). While some previous studies similarly reported a lack of strong inhibitory effects for the TT [76], others have documented more potent antibacterial activity, particularly against Gram-positive bacteria [1,4,77,78]. The limited efficacy observed in our study could be attributed to several factors, including the plant material’s specific geographic origin, harvesting time, and the anatomical parts used (flowers vs. leaves). Furthermore, the extraction solvent and methodology significantly influence the concentration and profile of bioactive secondary metabolites, which may account for the discrepancies observed between different studies.

This limited efficacy aligns with the findings of Razavi et al. [79], who similarly reported a lack of notable inhibitory effects for the TT against target bacterial strains. Conversely, some researchers have documented highly potent antibacterial activities. Arshad et al. [80] reported massive inhibition zones of 29 mm against *S. aureus* and 30 mm against *E. coli* using leaf extracts, and 16 mm and 21 mm using root extracts.

The profound discrepancy between the results obtained in the present study and the strong inhibition reported in the literature [1,4,77,78] can be primarily attributed to the specific anatomical parts utilized. While the current investigation evaluated extracts specifically derived from the floral parts of the plant, the literature clearly indicates that leaves generally constitute a much more potent source of antibacterial constituents than other organs. Furthermore, factors such as the specific geographical origin (the analyzed samples originating from İzmit, Türkiye, compared to those from Iran or Iraq), environmental conditions, harvesting time, and the choice of extraction solvent significantly dictate the concentration and profile of bioactive secondary metabolites. Thus, while the floral ethanol extract evaluated herein possesses mild inhibitory potential, optimizing plant source selection and extraction methodologies remains critical for therapeutic applications.

## 3. Materials and Methods

### 3.1. Chemicals, Reagents, and Instruments

All chemicals, reagents, and enzymes used in this study were of analytical grade. α-Glucosidase (EC 3.2.1.20) from Saccharomyces cerevisiae (Type I, C5003), acetylcholinesterase (AChE; EC 3.1.1.7) from Electrophorus electricus (Type VI-S, C3389), and butyrylcholinesterase (BChE; EC 3.1.1.8) from equine serum (C7512) were purchased from Sigma-Aldrich (St. Louis, MO, USA). Tacrine and acarbose were used as reference inhibitors in cholinesterase and α-glucosidase inhibition assays, respectively. All other analytical-grade chemicals and reagents were obtained from commercial suppliers.

Phenolic compounds were identified using liquid chromatography–tandem mass spectrometry (LC–MS/MS). Absorbance measurements for antioxidant and enzyme inhibition assays were performed using a double-beam UV–Vis spectrophotometer (UNICO, SQ4802, Franksville, WI, USA).

### 3.2. Plant Materials

The plant material of *Tribulus terrestris* L. was collected from Ambarcı Village, Kandıra district, İzmit Province, located in the Marmara Region of Türkiye, during May–June 2025 (Latitude: 40.891533, Longitude: 30.184597, altitude: 300 m). The taxonomic identification and authentication of the plant material (flower and leaf together) were performed by Prof. Dr. Mustafa Korkmaz from the Department of Biology, Erzincan Binali Yıldırım University, Türkiye, and the species was confirmed as *Tribulus terrestris* L. (TT) (Figure 2). The collected plant material was washed thoroughly with running tap water three to four times and then shade-dried under ambient laboratory conditions. After complete drying, the plant material was ground into a fine powder using a mechanical grinder, transferred into airtight sample containers, and stored at 4 °C until further analysis. All experiments were performed in triplicate to ensure reproducibility.

### 3.3. Preparation of Plant Extract

The extraction procedure was conducted with minor modifications based on the method previously described by Atmani et al. [80]. The dried plant material (TT) was mixed with ethanol at a 1:10 (*w*/*v*) ratio and subjected to continuous agitation for 24 h to facilitate extraction. The resulting extract was filtered through Whatman No. 1 filter paper, after which the solvent was removed using a rotary evaporator. The obtained samples were stored in sealed containers at 4 °C until further analysis. LC–MS/MS results were evaluated considering the total dry matter content of the samples [81].

### 3.4. Phenolic Compound Analysis

The phenolic profile of TT was determined by LC–MS/MS using a Shimadzu Nexera series UHPLC system coupled to a tandem mass spectrometer. Method validation for the simultaneous analysis of twenty phenolic compounds was conducted at the Central Research Laboratory of Harran University. The analytical setup included LC-30AD dual pumps, a DGU-20A3R degasser, a CTO-10ASVP column oven, and a SIL-30AC autosampler. Chromatographic separation was achieved on a C18 Inertsil ODS-4 analytical column (3.0 mm × 100 mm, 2 μm particle size). The column temperature was maintained at 40 °C, and gradient elution was performed using mobile phase A (water containing 0.1% formic acid) and mobile phase B (methanol containing 0.1% formic acid). The injection volume was set to 4 μL, and the flow rate was kept constant at 0.5 mL min^−1^. Mass spectrometric detection was performed on a Shimadzu LC-MS 8040 triple quadrupole instrument equipped with an electrospray ionization (ESI) source, operating in both positive and negative ion modes. Data acquisition and processing were performed using LabSolutions software (Shimadzu, Kyoto, Japan), and quantification was carried out in multiple reaction monitoring (MRM) modes [82]. Before LC–MS/MS analysis, the dried ethanolic extract was accurately weighed and dissolved in methanol–water (1:1, *v*/*v*) to obtain a stock solution. The solution was vortexed and sonicated to ensure complete homogenization. An aliquot of the stock solution was then diluted with methanol–water (1:1, *v*/*v*) to bring the analyte responses within the validated calibration range of 1–2000 µg L^−1^. The dilution strategy was selected to keep the analyte responses within the validated calibration range and to avoid detector saturation. After preliminary injections, the same dilution protocol was applied uniformly to all LC–MS/MS analytical solutions prepared from the TT ethanolic extract; no analyte-specific or case-by-case dilution adjustment was applied during quantitative reporting. The final diluted solution was filtered through a 0.45 µm PTFE membrane filter, transferred into amber LC vials, and injected into the LC–MS/MS system. All LC–MS/MS concentration values were calculated for the final analytical solution and therefore do not represent dry-weight-normalized concentrations of the original plant material.

Calibration curves were constructed using authentic standards over the concentration range of 1–2000 µg L^−1^. LOD and LOQ values were generated using Shimadzu Lab Solutions LCMS software during method validation. For each analyte, the software calculated the chromatographic signal-to-noise ratio from the integrated MRM peak relative to the local baseline noise under identical acquisition and integration parameters. LOD and LOQ values were assigned according to the software-based signal-to-noise approach, corresponding to the commonly applied criteria of approximately S/N ≥ 3 for LOD and S/N ≥ 10 for LOQ. Therefore, LOD and LOQ values were not manually estimated but were obtained from the software-generated validation outputs for each analyte. Analytes producing detectable signals above the LOD but below the corresponding LOQ were reported as DNQ and were not included in quantitative ranking or comparative interpretation. Accordingly, kaempferol was consistently classified as DNQ because its signal was detectable but below the corresponding LOQ. Therefore, it was excluded from the list of quantified compounds. Compound assignment was not based solely on MRM transitions. The retention time of each analyte was confirmed using individually injected authentic standards analyzed under the same chromatographic conditions. Particular attention was given to structurally related compounds and positional isomers that may share the same nominal MRM transition. For example, 4-hydroxybenzoic acid and salicylic acid were evaluated using their standard-derived retention times under identical chromatographic conditions. In addition, Q1 scan mass spectra of the individual authentic standards were examined as supporting evidence for their ion abundance patterns. Quercetin and butein were also evaluated with particular caution because of their close retention behavior; however, they were distinguished using their individually injected authentic standards and compound-specific precursor/product ion transitions. Thus, the assignment of these compounds was based on a combination of retention time matching, MRM transition monitoring, and standard-based spectral verification. Considering the complexity of plant matrices, these assignments should be interpreted as standard-supported targeted LC–MS/MS identifications rather than de novo structural elucidation; therefore, potential matrix-related co-elution or matrix interference cannot be completely excluded without additional orthogonal confirmation such as high-resolution MS/MS.

### 3.5. Antioxidant Activity

#### 3.5.1. Total Reducing Capacity

The metal-reducing ability of TT extracts was evaluated using a modified version of the method described by Elmastaş et al. [83]. Plant extracts at varying concentrations (10, 20, and 40 μg mL^−1^) were combined with 2.5 mL of phosphate buffer (0.2 M, pH 6.6) and 2.5 mL of 1% potassium ferricyanide [K_3_Fe(CN)_6_]. The resulting mixtures were incubated at 50 °C for 20 min. After incubation, 2.5 mL of 10% trichloroacetic acid (TCA) was added, and the reaction mixtures were centrifuged at 3000 rpm for 10 min. Subsequently, 2.5 mL of the supernatant was mixed with 2.5 mL of distilled water and 0.25 mL of 0.1% FeCl_3_ solution. The absorbance was then measured at the appropriate wavelength after color development. The absorbance of the supernatants was measured at 700 nm. Reducing power was expressed in terms of absorbance values, and the findings were compared with those obtained from standard antioxidant compounds [84].

#### 3.5.2. Cu(II) Ion Reducing Capacity (CUPRAC)

The cupric ion reducing antioxidant capacity (CUPRAC) assay, based on the reduction of the Cu(II)–neocuproine (Cu(II)–Nc) complex to the Cu(I)–neocuproine (Cu(I)–Nc) form, was carried out according to the method reported by Apak et al. [85] and Gülçin [86]. Briefly, 1 mL of CuCl_2_ solution (0.01 M), 1 mL of neocuproine (2,9-dimethyl-1,10-phenanthroline), and 1 mL of ammonium acetate (NH_4_Ac) buffer were added to a test tube and mixed thoroughly using a vortex mixer. Subsequently, plant extracts at different concentrations (10, 20, and 40 μg mL^−1^) were introduced, and the final volume was adjusted to 4 mL with ultrapure water. After incubation at room temperature for 30 min, the absorbance was measured at 450 nm. The increase in absorbance resulting from the reaction indicates an enhanced Cu(II) ion reducing capacity. The results were expressed as absorbance values and compared with those of standard antioxidant compounds.

#### 3.5.3. DPPH Radical Scavenging Activity

The free radical scavenging capacity of TT extracts, along with reference antioxidants, was assessed using the method originally described by Blois [87]. For this purpose, a 0.1 mM DPPH solution was prepared in methanol. Aliquots of the extract at concentrations of 10, 20, and 40 μg mL^−1^, obtained from stock solutions, were mixed with 1 mL of the DPPH solution and diluted to a final volume of 3 mL using ethanol. The mixtures were vortexed thoroughly and incubated in the dark for 30 min. Absorbance values were then recorded at 517 nm using a spectrophotometer. The results were expressed as the percentage of radical scavenging activity.

#### 3.5.4. ABTS Radical Scavenging Activity

This assay is based on the discoloration of the ABTS^+^ radical cation upon interaction with antioxidant compounds in the extracts, as described by Re et al. [88]. An ABTS solution (2 mmol L^−1^) was reacted with potassium persulfate (K_2_S_2_O_8_, 2.45 mmol L^−1^) to generate the radical cation. The resulting mixture was incubated in the dark at room temperature for 14 h to allow complete formation of the ABTS^+^ radical. Before analysis, the ABTS^+^ solution was diluted with sodium phosphate buffer (0.1 mol L^−1^, pH 7.4) to achieve an absorbance of 0.750 ± 0.025 at 734 nm. Subsequently, aliquots of extract solutions at concentrations of 10, 20, and 40 μg mL^−1^ were prepared and adjusted to a final volume of 3 mL using the same phosphate buffer. Then, 1 mL of the prepared ABTS^+^ solution was added to each extract sample and mixed thoroughly using a vortex. The absorbance was recorded at 734 nm with a spectrophotometer. The scavenging activity was calculated and expressed as a percentage of radical inhibition.

### 3.6. Cholinesterase Inhibition Assay

The inhibitory activity of the TT ethanol extract against acetylcholinesterase (AChE; EC 3.1.1.7, from Electrophorus electricus, Type VI-S, C3389) and butyrylcholinesterase (BChE; EC 3.1.1.8, from equine serum, C7512) was evaluated using the spectrophotometric assay described by Ellman et al. with minor modifications [89]. The reaction mixture was prepared using Tris-HCl buffer (pH 8.0), cholinesterase enzyme solution, 5,5′-dithio-bis (2-nitrobenzoic acid) (DTNB), and substrate solution. Acetylthiocholine iodide was used as the substrate for AChE, whereas butyrylthiocholine iodide was used for BChE. Different concentrations of the TT ethanol extract were added to the reaction medium to determine the inhibitory activity. The reaction mixtures were incubated at 25–30 °C for a specified time to allow enzymatic hydrolysis. The formation of the 5-thio-2-nitrobenzoate anion, produced by the reaction of DTNB with thiocholine released during substrate hydrolysis, was measured at 412 nm using a UV–Vis spectrophotometer. The inhibition observed with 20% DMSO alone (used as the negative control) was subtracted from the total inhibition values, ensuring that only the inhibition due to the tested compounds was considered in the analysis. To assess possible interference from the intrinsic color of the extracts, sample blanks containing the extract and all assay components except the enzyme were prepared, and their absorbance values were recorded. These values were used for background correction where necessary. No significant absorbance interference attributable to extract color was detected at the wavelengths used for the assays.

Tacrine, a known cholinesterase inhibitor, was used as the positive control and tested under the same experimental conditions as the extract. Enzyme inhibition was calculated as a percentage relative to the control group. The concentrations required to inhibit 50% of AChE and BChE activities (IC_50_) were determined from activity (%)–concentration curves [90,91]. All assays were performed in triplicate (*n* = 3), and the results were expressed as mean ± standard deviation (SD).

### 3.7. α-Glucosidase Inhibition Analysis

The α-glucosidase inhibitory activity of the TT ethanol extract was determined according to the method described by Tao et al. [92] with minor modifications. The extract was initially dissolved in 20 µL DMSO, and the final volume was adjusted to 1 mL with deionized water to prepare the stock solution. The obtained stock solution was subsequently diluted 10-fold with deionized water to obtain the required test concentrations. For the enzyme inhibition assay, 200 µL of phosphate buffer (0.1 M, pH 6.9), 10 µL of α-glucosidase enzyme solution (0.11 U/mL; from *Saccharomyces cerevisiae*, Type I, C5003), and different concentrations of TT extract or acarbose solution were mixed and pre-incubated at 35 °C for 10 min. The enzymatic reaction was initiated by adding 20 µL of p-nitrophenyl-α-D-glucopyranoside (p-NPG, 5 mM), and the reaction mixtures were further incubated at 35 °C for 20 min. The absorbance was measured at 405 nm using a UV–Vis spectrophotometer [93]. The inhibition observed with 20% DMSO alone (used as the negative control) was subtracted from the total inhibition values, ensuring that only the inhibition due to the tested compounds was considered in the analysis.

The IC_50_ values were calculated from concentration–inhibition curves [90,91,93]. Acarbose was used as the positive control. All assays were performed in triplicate (*n* = 3), and the results were expressed as mean ± standard deviation (SD).

### 3.8. Determination of the Antibacterial Activity of TT Extract

#### 3.8.1. Preparation of Bacterial Cultures

The antibacterial potential of TT extracts was evaluated using *E. coli* (ATCC 25922), *S. aureus* (ATCC 25923), and *S. typhimurium* (ATCC 14028) as test microorganisms. These reference strains were obtained from the culture collection of the Department of Food Engineering, Faculty of Agriculture, Atatürk University (Erzurum, Türkiye), where they were preserved at −80 °C until further analysis.

Before experimentation, the cultures were reactivated in Trypticase Soy Yeast Extract (TSYE) broth and incubated at 35–37 °C for 24 h. Subsequently, the turbidity of the bacterial suspensions was adjusted to match the 0.5 McFarland standard, corresponding to approximately 1 × 10^8^ CFU mL^−1^ [94].

#### 3.8.2. Minimum Inhibitory Concentration (MIC) Assay

For the MIC determination, TT extracts were prepared at a concentration of 312 μg mL^−1^ (*w*/*v*) in 35% dimethyl sulfoxide (DMSO). The resulting solutions were sterilized by filtration through 0.45 μm Millipore membranes and stored in sterile 1.5 mL Eppendorf tubes at 4 °C until further use.

An aliquot of 10 μL from each bacterial inoculum was transferred into microplate wells, and the plant extract was serially diluted in nutrient broth (NB) to obtain concentrations of 312, 156, 78, 39, 19.5, and 9.75 mg mL^−1^. A sterile DMSO solution served as the negative control. The prepared microplates were incubated at 35–37 °C for 24 h. The presence of turbidity in the wells was interpreted as bacterial growth, whereas clear wells indicated inhibition. At the end of the incubation period, all wells were visually examined for growth or absence thereof. Each experiment was performed in triplicate [74].

### 3.9. Statistical Analysis

All experimental data were analyzed using GraphPad Prism (version 6; GraphPad Software, La Jolla, CA, USA). Results were expressed as mean ± standard deviation (95% confidence interval). Statistical differences among datasets were considered significant at *p* ≤ 0.05. All experiments were performed in triplicate and independently repeated three times.

## 4. Conclusions

The present study demonstrated that the TT is a valuable source of biologically active phytochemicals with diverse pharmacological properties. Comparative analyses of plant parts (flower and leaf) revealed marked variations in both phytochemical composition and biological activity. The antibacterial and antioxidant assays revealed that leaf extracts showed greater efficiency in biological action, indicating that the leaves may constitute a more abundant source of bioactive nutrients. The findings further confirmed that TT possesses a broad spectrum of bioactivities, including antioxidant, antibacterial, antiproliferative, and anticholinesterase effects. The strong association between its phenolic-rich composition and biological efficacy highlights the potential role of these compounds in mediating the observed activities. Among the plant parts investigated, the leaves emerged as the most promising source of bioactive constituents, suggesting their suitability for future phytopharmaceutical applications.

The biological activity of vanillic acid was not validated using the isolated pure compound, and the relationship between the identified constituents and the observed bioactivities was not directly established. Further studies are needed to confirm constituent-specific biological activities.

Notably, the inhibitory activity observed against *E. coli* provides experimental evidence supporting the traditional use of Iraqi TT in the treatment of urinary tract infections. This result reinforces the ethnopharmacological relevance of the plant and indicates its potential as a natural antimicrobial agent.

Overall, the study provides comprehensive evidence that TT may serve as a promising candidate for the development of natural products intended for pharmaceutical, nutraceutical, food preservation, and feed additive applications. Nevertheless, further investigations involving the isolation of active constituents, mechanistic studies, and in vivo evaluations are warranted to validate its efficacy and safety and to facilitate its potential therapeutic utilization.

## Figures and Tables

**Figure 1:**
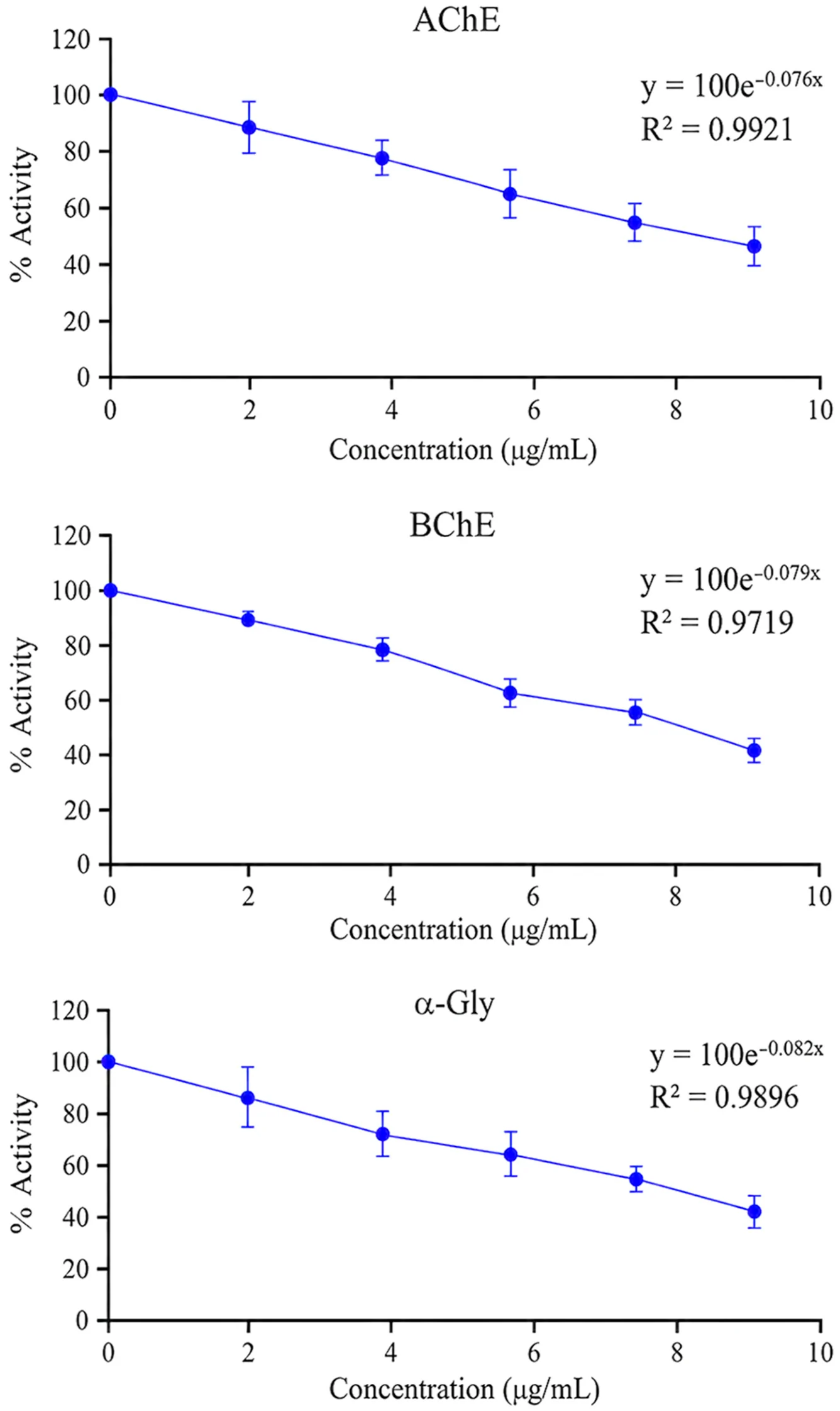
Dose-dependent inhibitory effects of the TT ethanol extract on AChE, BChE, and α-Glucosidase enzymes. Experiments were performed in 3 replicates.

**Figure 2:**
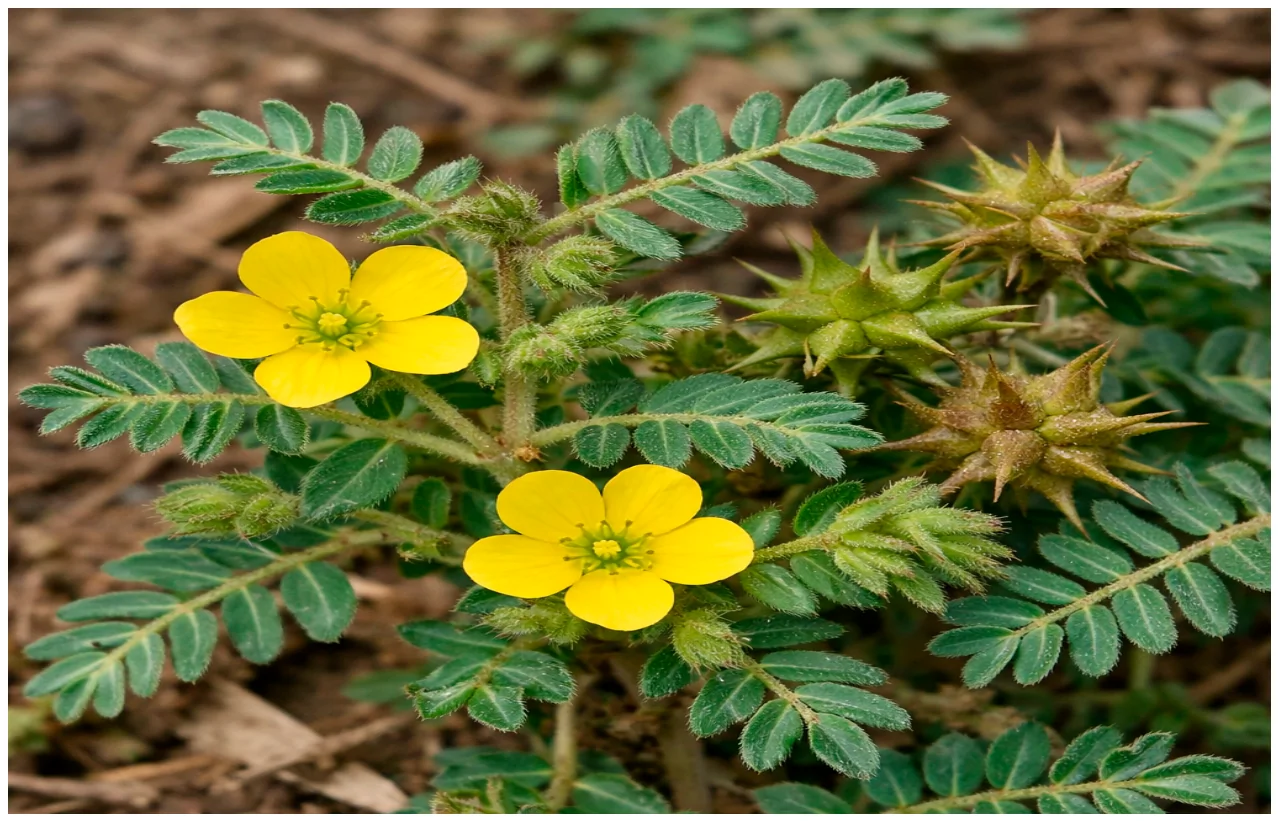
Image of *Tribulus terrestris*.

**Table 1:** LC–MS/MS determination of phytochemical and phenolic compounds in the final ethanolic extract solution of *Tribulus terrestris* (TT).

Standard Compounds	^a^ MRM	^b^ RSD %	^c^ LOD/LOQ (μg L^−1^)	Recovery * (%)	^d^ RT	^e^ R^2^	Equation	Concentration (µg L^−1^) **
Quercetin	301.1 > 151	1.36	22.5/25.7	100.1	6.091	0.999	Y = (13.7831)X + (−146.951)	116.59
Acetohydroxamic Acid	76.10 > 43.10	8.2	2.8/8.2	100.0	1.986	0.999	Y = (150.982)X + (23.1833)	317.58
Catechin hydrate	291.10 > 139.00	2.36	8.2/11.4	99.4	4.958	0.999	Y = (79.2933)X + (−2406.22)	25.76
Vanillic Acid	168.80 > 93.00	6.2	125.5/142.2	100.1	6.026	0.998	Y = (48.0522)X + (−876.904)	1080.74
Resveratrol	229.10 > 135.00	1.31	9.0/13.6	99.8	5.713	0.998	Y = (46.4361)X + (−1314.61)	502.34
Fumaric Acid	115.20 > 71.00	0.47	25.2/31.3	99.7	3.674	0.999	Y = (20.2986)X + (−762.592)	439.03
Gallic acid	169.20 > 125.00	1.36	0.90/1.6	100.0	4.134	0.999	Y = (65.3835)X + (−2699.84)	N.D.
Caffeic Acid	179.20 > 135.00	1.37	6.3/10.7	100.9	5.283	0.996	Y = (124.785)X + (−487.132)	35.17
Phloridzin dihydrate	435.00 > 273.10	5.64	61.0/207.0	100.0	5.646	0.999	Y = (33.4069)X + (−1396.90)	DNQ
Oleuropein	539.10 > 377.20	6.94	0.05/1.0	99.7	5.643	0.999	Y = (25.9240)X + (−558.916)	N.D.
Ellagic Acid	300.90 > 145.10	8.56	0.101/0.333	100.2	5.895	1.000	Y = (5.25903)X + (−1167.31)	N.D.
Myricetin	317.10 > 150.90	0.79	55.4/59.6	99.9	5.858	0.999	Y = (37.0934)X + (2684.23)	N.D.
Protocatechuic acid	181.20 > 108.00	1.29	30.3/35.4	101.1	5.875	0.994	Y = (526.954)X + (23026.1)	N.D.
Butein	271.10 > 135.00	1.45	22.7/28.6	96.0	6.084	0.999	Y = (49.3543)X + (367.917)	92.42
Naringenin	271.10 > 150.90	2.05	5.4/6.4	99.8	6.104	0.996	Y = (317.241)X + (33733.3)	N.D.
Luteolin	285.20 > 132.90	0.57	0.5/2.5	100.7	6.190	0.998	Y = (34.6668)X + (3721.79)	N.D.
Kaempferol	285.10 > 116.90	1.44	206.6/214.3	99.9	6.288	0.999	Y = (2.63905)X + (−206.494)	DNQ
Alizarin	239.20 > 210.90	3.51	65.2/77.5	96.6	6.800	0.998	Y = (3.97487)X + (1614.23)	N.D.
4-Hydroxybenzoic Acid	137.20 > 93.00	1.54	30.5/40.25	99.6	6.130	0.999	Y = (735.804)X + (−498.102)	108.89
Salicylic acid	137.20 > 93.00	1.24	4.2/7.6	100.9	6.104	0.999	Y = (746.369)X + (6072.41)	104.24

^a^ MRM: Multiple Reaction Monitoring, ^b^ RSD: Relative standard deviation, ^c^ LOD/LOQ (µg/L): limit of detection/limit of quantitation, ^d^ RT: Retention time, ^e^ R^2^: Determination coefficient, N.D.: Not detected. DNQ: detected but not quantified; the analyte signal was detected but was below the corresponding LOQ. * Recovery values are expressed as percentages and were calculated as follows: recovery (%) = (measured concentration/spiked concentration) × 100. ** Concentration values expressed as µg L^−1^ refer to the final LC–MS/MS analytical solution prepared from the ethanolic extract and do not represent dry-weight-normalized contents of the original plant material.

**Table 2:** Inhibition effect of the TT extract on radical scavenging and metal reducing activity.

Antioxidants	DPPH ^a^ (0.2 μg mL^−1^)	ABTS ^a^ (0.2 μg mL^−1^)	FRAP Assay ^b^ (0.2 μg mL^−1^)	CUPRAC Assay ^b^ (0.2 μg mL^−1^)
*Tribulus terrestris*	6.224 ± 0.04	10.386 ± 0.82	0.136 ± 0.01	0.296 ± 0.02
BHA	71.819 ± 3.82	83.668 ± 5.62	0.454 ± 0.03	-
BHT	46.329 ± 2.67	48.353 ± 2.48	0.624 ± 0.04	-
Trolox	81.187 ± 5.42	80.063 ± 6.75	0.252 ± 0.01	0.516 ± 0.03

Standard antioxidants (BHA, butylated hydroxyanisole; BHT, butylated hydroxytoluene; trolox). ^a^ Values are expressed as percent radical scavenging activity. ^b^ Values are expressed as absorbance. High absorbance indicates high metal reduction capacity.

**Table 3:** Inhibitory activities of *Tribulus terrestris* ethanol extract for AChE, BChE, and α-Glucosidase enzymes.

Inhibitor	AChE IC_50_ (μg mL^−1^)	R^2^	BChE IC_50_ (μg mL^−1^)	R^2^	α-Glucosidase IC_50_ (μg mL^−1^)	R^2^
*Tribulus terrestris*	9.11 ± 2.12 ^b^	0.992	8.77 ± 0.62 ^b^	0.972	8.45 ± 0.45 ^b^	0.989
Tacrine	3.89 ± 0.28 ^a^	0.998	3.36 ± 0.32 ^a^	0.959	-	-
Acarbose	-	-	-	-	4.00 ± 0.35 ^a^	0.998

IC_50_ results are expressed as the mean ± SD (*n* = 3). Mean values followed by different letters in the same column are significantly different (*p* ≤ 0.05).

**Table 4:** Antibacterial effect of *Tribulus terrestris* extract (Inhibition zone, mm).

Sample (6.24 mg/Disk)	Concentration	Inhibition Zone Diameter (mm) *S. aureus*	Inhibition Zone Diameter (mm) *E. coli*	Inhibition Zone Diameter (mm) *S. typhimurium*
*Tribulus terrestris*	312 μg mL^−1^	4	5	8
Control (Ciprofloxacin)	5 μg	19	21	18

**Table 5:** MIC results of *Tribulus terrestris* extract (mg/mL).

*Tribulus terrestris*	Concentration (μg/mL)	Inoculum Amount (μL)	*S. aureus*	*E. coli*	*S. typhimurium*
	312	10	-	-	-
	156	10	+	+	-
	78	10	+	+	+
	39	10	+	+	+
	20	10	+	+	+
	10	10	+	+	+
Medium + Inoculum	0	10	+	+	+
Medium + Solvent (DMSO)	0	10	+	+	+
Medium	0	0	-	-	-

(+): Growth, (-): No Growth.

## Data Availability

The original contributions presented in this study are included in the Article/Supplementary Materials. Further inquiries can be directed to the corresponding authors.

## Supplementary Materials

The following supporting information can be downloaded at: https://www.mdpi.com/article/10.3390/molecules31193370/s1, Figure S1: Representative LC-MS/MS chromatogram for standard phenolic compounds.

## Abbreviations

The following abbreviations are used in this manuscript:

TT	*Tribulus terrestris*
LC-MS/MS	Liquid Chromatography–Tandem Mass Spectrometry
DPPH	DPPH2,2-Diphenyl-1-picrylhydrazyl
FRAP	Ferric Reducing Antioxidant Power
ABTS	2,2′-Azino-bis(3-ethylbenzothiazoline-6-sulfonic acid)
AChE	Acetylcholinesterase
BChE	Butyrylcholinesterase
DTNB	(5,5′-Dithiobis-(2-nitrobenzoic acid))
p-NPG	p-nitrophenyl-D-glucopyranoside
ATCC	American Type Culture Collection
CFU/mL	Colony-forming units per milliliter
MIC	Minimum Inhibitory Concentration
McF	McFarland
