# Supplementary material for: Assessment of Antioxidant Capacity, Phytochemical Phenolic Composition, Cholinesterase and α-Glucosidase Inhibitory Activities, and Antimicrobial Potential of Tribulus terrestris
Source: Molecules. 2026 Sep 22;31(19):3370. doi: 10.3390/molecules31193370 (PMC13665852; doi:10.3390/molecules31193370)
Supplement: Supplementary file 1 [file molecules-31-03370-s001.zip › molecules-4398721-supplementary.pdf]

***Supplementary Material***

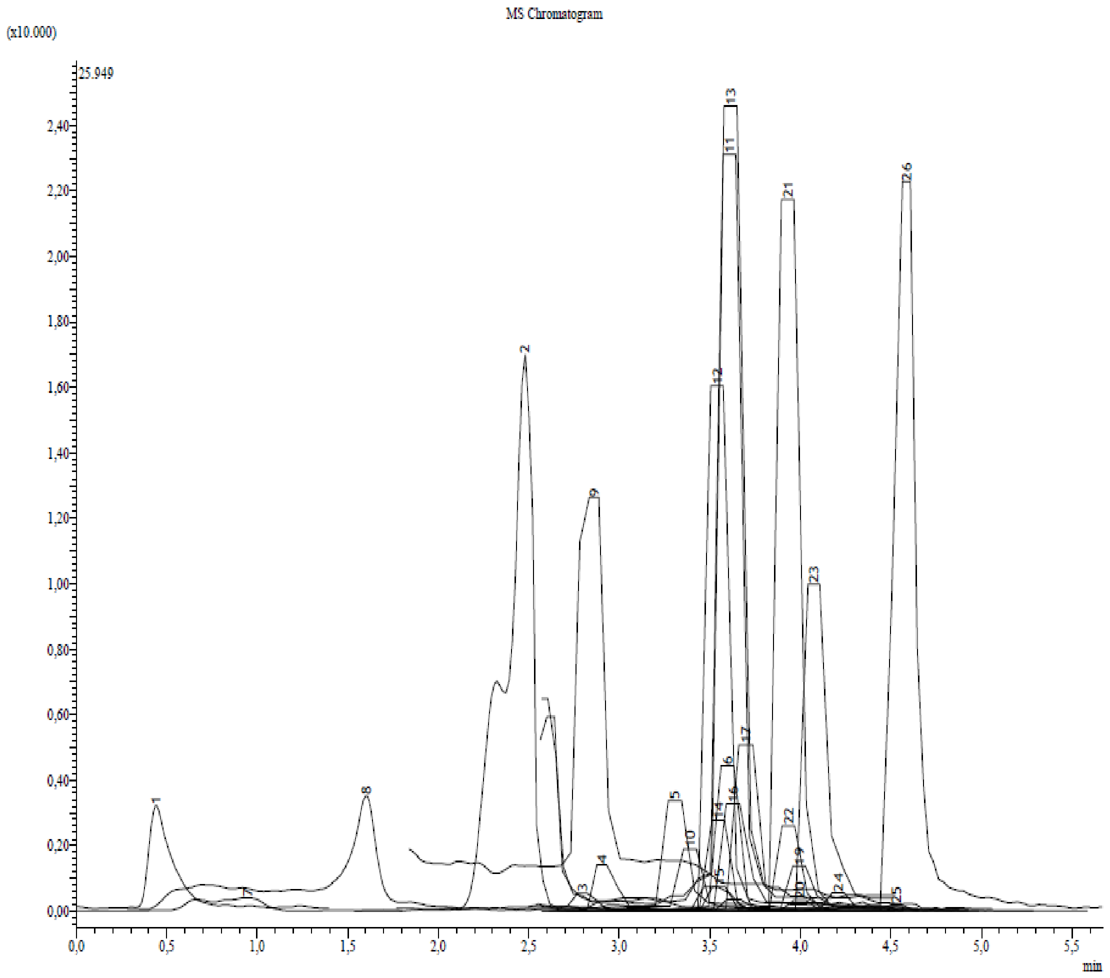


**Figure S1.** Representative LC-MS/MS chromatogram for standard phenolic compounds.

| | **Name** |
|---|---|
| 1 | Acetohydroxamic Acide_poz |
| 2 | Catechinhyrate_pos |
| 3 | Vanilic Acide_poz |
| 4 | Syringic acid_pos |
| 5 | Thymoquinone_poz |
| 6 | Resveratrol_pos |
| 7 | Fumaric Acid _neg |
| 8 | Gallic acid_neg |
| 9 | Caffeic acid_neg |
| 10 | Hydoxycinamic_neg* |
| 11 | 4-Hydroxybenzoic acid _neg |
| 12 | Protocatechuic acid _neg |
| 13 | Salicylic acid_neg |
| 14 | Oleuropein_neg |
| 15 | Phloridzindyhrate_neg |
| 16 | 2-hyroxy1.4nph_neg |
| 17 | Myricetin_neg |
| 18 | Ellagic acid_neg |
| 19 | Quercetin_neg |
| 20 | Bütein_neg |
| 21 | Naringenin_neg |
| 22 | Silymarin_neg |
| 23 | luteolin_neg |
| 24 | Kaempferol_neg |
| 25 | Alizarin_neg |
| 26 | Curmin_neg |
| | |
